# Predicting Drug–Gene–Disease Associations by Tensor Decomposition for Network-Based Computational Drug Repositioning

**DOI:** 10.3390/biomedicines11071998

**Published:** 2023-07-14

**Authors:** Yoonbee Kim, Young-Rae Cho

**Affiliations:** 1 Division of Software, Yonsei University Mirae Campus, Wonju-si 26493, Gangwon-do, Republic of Korea; yoonbee7@yonsei.ac.kr; 2 Division of Digital Healthcare, Yonsei University Mirae Campus, Wonju-si 26493, Gangwon-do, Republic of Korea

**Keywords:** drug repositioning, drug–disease associations, heterogeneous networks, drug networks, disease networks

## Abstract

Drug repositioning offers the significant advantage of greatly reducing the cost and time of drug discovery by identifying new therapeutic indications for existing drugs. In particular, computational approaches using networks in drug repositioning have attracted attention for inferring potential associations between drugs and diseases efficiently based on the network connectivity. In this article, we proposed a network-based drug repositioning method to construct a drug–gene–disease tensor by integrating drug–disease, drug–gene, and disease–gene associations and predict drug–gene–disease triple associations through tensor decomposition. The proposed method, which ensembles generalized tensor decomposition (GTD) and multi-layer perceptron (MLP), models drug–gene–disease associations through GTD and learns the features of drugs, genes, and diseases through MLP, providing more flexibility and non-linearity than conventional tensor decomposition. We experimented with drug–gene–disease association prediction using two distinct networks created by chemical structures and ATC codes as drug features. Moreover, we leveraged drug, gene, and disease latent vectors obtained from the predicted triple associations to predict drug–disease, drug–gene, and disease–gene pairwise associations. Our experimental results revealed that the proposed ensemble method was superior for triple association prediction. The ensemble model achieved an AUC of 0.96 in predicting triple associations for new drugs, resulting in an approximately 7% improvement over the performance of existing models. It also showed competitive accuracy for pairwise association prediction compared with previous methods. This study demonstrated that incorporating genetic information leads to notable advancements in drug repositioning.

## 1. Introduction

Drug repositioning, which identifies additional therapeutic indications for drugs that have already been commercialized, has gained attention owing to its ability to significantly reduce the time and cost required to develop novel drugs [1]. This approach is appealing to the pharmaceutical industry because there is a substantial pool of drugs that have been developed and approved but are underutilized. In particular, computational methods have shown promise in this field by inferring potential associations between drugs and diseases efficiently [2]. For effective repositioning of drugs, it is crucial to identify the proteins targeted by drugs or genes causing diseases, as drugs function by inhibiting or activating specific genes. Computational techniques extensively analyze omics data, including drugs, diseases, disease-causing genes, and drug–targeted proteins, to narrow down the list of candidates for experimental drug repositioning. The rapid accumulation of such data has been facilitated by advancements in genomics, proteomics, and systems biology.

Initially, the early research in the domain of computational drug repositioning primarily focused on uncovering the interactions between a drug and its molecular targets [3,4,5]. Evidence of drug–target interactions (DTIs) serves as a significant clue for drug repositioning based on the underlying assumption that multiple drugs interact with multiple targets [6]. Previous DTI prediction methods have often explored networks such as drug–target bipartite networks [7,8] to infer new targets for each drug. For example, BLM-NII [9] employed a neighbor-based interaction-profile inference (NII) algorithm to predict DTIs within the bipartite local model (BLM), which cannot be learned in the absence of known interactions. WNN-GIP [10] constructed interaction profiles for drugs and proteins using the weighted nearest neighbor (WNN) algorithm, enhanced interaction evidence through the Gaussian interaction profile kernel (GIP), and predicted DTIs using normalized least-squares (RLS). NetLapRLS [11] adopted the Laplace regularized least-squares method for DTI prediction. MSCMF [12] integrated multiple drug and disease similarity matrices, updating the weights for low-rank similarity matrices of drugs and targets using the cross-alternative least-squares algorithm. BRDTI [13] extended the Bayesian personalized ranking matrix factorization method to match drugs and targets even in the absence of positive samples, such as new drugs. More advanced methods employed complex heterogeneous networks, incorporating both drugs and target proteins, for predicting DTIs [14,15,16].

Recent disease studies that aim to identify disease-causing genes have revealed an increasing trend towards network-based analysis. Networks have become essential tools for prioritizing genes associated with a disease [17]. Numerous network-based methods have been proposed for predicting disease–gene associations [18,19]. These methods operate under the assumption that genes responsible for the same disease tend to be located in close proximity within a disease network. For example, PRINCE [20] applied a network propagation algorithm to a disease–gene heterogeneous network to predict disease–gene associations. A homogeneous network comprises nodes of a single type and their connections, whereas a heterogeneous network is comprised of two or more types of nodes and their connections. HerGePred [21] performed a random walk with restart on a disease–gene heterogeneous network, incorporating network embeddings, such as node2vec, to obtain low-dimensional vectors and reconstruct the networks based on their similarities.

Most computational drug repositioning approaches for predicting drug–disease associations also address the challenges posed by heterogeneous networks that incorporate various features of drugs and diseases [22]. Network-based methods can be categorized into three groups based on their key techniques. The first group applies graph mining algorithms, such as random walk, network propagation, path search, and clustering, to detect putative associations between drug and disease pairs that are not directly linked in a heterogeneous network. For example, TL-HGBI [23] integrated drug, disease, and gene similarities into a three-layer heterogeneous network and inferred drug–disease associations using an information propagation algorithm. MBiRW [24] predicted drug–disease associations using a bi-random walk algorithm on a heterogeneous network constructed using drug and disease similarities. DR-IBRW [25] employed dual random work based on the concept that each node in the network has a distinct walk length to facilitate the dissemination of information.

The second group of drug–disease association prediction approaches uses deep learning algorithms, including autoencoders and graph convolutional networks, to extract network features and construct predictive models. For deepDR [26], common features from nine drug similarity networks were extracted using a multi-modal deep autoencoder. These features, along with drug–disease associations, were then input into a collective variable autoencoder to infer novel indications for a given drug. ANMF [27] predicted associations by leveraging the drug and disease features extracted through an autoencoder by multiplying them.

The last group of approaches to predict drug–disease associations employs matrix factorization or matrix completion techniques, which are based on collaborative filtering methods that analyze correlations between entities. A collection of drug–disease associations is represented in a matrix form, and matrix factorization is applied to decompose it into two matrices of latent features representing drugs and diseases. The predictive scores for drug–disease associations are then computed by multiplying the two latent feature matrices. For example, MSBMF [28] performed matrix decomposition by combining multiple drug–drug and disease–disease similarity networks to identify new indications. NMF-DR [29] constructed a heterogeneous drug–disease network and performed non-negative matrix decomposition. SCPMF [30] used each similarity as a constraint on drugs and viruses during matrix decomposition to predict associations between drugs and viruses. Instead of constructing a single heterogeneous network, OMC [31] adopted separate matrix completion techniques to handle drug and disease networks independently.

Most matrix factorization methods, as described above, focus primarily on utilizing drug and disease networks. However, considering that genes also interact closely with drugs and diseases, incorporating features of all three components can lead to more accurate outcomes not only in drug–disease association prediction, but also in DTI and disease–gene association prediction. Tensors are commonly used to integrate these components. Tensor decomposition, which extends the concept of matrix decomposition to multiple dimensions, can be applied to discover novel associations among these components [32]. For example, Wang et al. [33] constructed a drug–target-disease tensor by combining drug–disease, drug–target, and disease–target associations and predicted drug–target-disease associations through tensor decomposition. NTD-DR [34] performed non-negative tensor decomposition on a drug–target-disease tensor for drug repositioning. Non-negative tensor decomposition was enhanced with additional constraints based on similarities and associations between drugs, targets, and diseases. TDRC [35] placed constraints related to biological similarity during tensor decomposition to identify links between microRNAs and diseases.

In this article, we present a network-based approach for computational drug repositioning that leverages a drug–gene–disease tensor containing drug–disease, drug–gene, and disease–gene associations. We employed tensor decomposition to predict drug–gene–disease triple associations. Additionally, we can predict pairwise associations by constructing drug–disease, drug–gene, and disease–gene matrices from latent factor matrices obtained through tensor decomposition.

However, tensor decomposition alone fails to capture the complex structure of drug–gene–disease associations and does not fully utilize the additional information available on drugs, genes, and diseases. This limitation arises because the tensor decomposition yields latent factor matrices through linear combinations. To overcome these limitations, neural network models can be employed because they excel at modeling nonlinear relationships and extracting diverse features from multiple datasets. Therefore, we propose a novel method for predicting drug–gene–disease triple associations by extending tensor decomposition to a nonlinear model. This method incorporates neural network models to handle the features from each dimension effectively. The aim of this study was to assess the degree of accuracy enhancement achieved by using predicted triple associations for computational drug repositioning.

## 2. Method

### 2.1. Preliminaries

A tensor is a multidimensional array representing different types of data, with each dimension corresponding to a specific type. It can take various forms such as a scalar when it has zero dimensions, a vector when it has one dimension, and a matrix when it has two dimensions. Tensor decomposition is a generalized technique that extends the decomposition of two-dimensional matrices into higher-dimensional tensors. CANDECOMP/PARAFAC (CP) decomposition [36] is the most widely used tensor decomposition model. The rank-1 tensor is defined as the outer product of these vectors. Then, CP decomposition factorizes tensor X into a sum of rank-1 tensors, as follows:

(1) $$X\approx \sum_{r=1}^{R}a_{r}\times b_{r}\times c_{r};$$

the symbol × denotes the outer product of vectors *a*, *b*, and *c*. Also, $a_{r}\in R^{I}$, $b_{r}\in R^{J}$, and $c_{r}\in R^{K}$ in the range of 1≤r≤R, where *I*, *J*, and *K* represent the dimensions of the tensors. *R* is a positive number that signifies the tensor rank. The rank of the tensor X is the minimum number of rank-1 tensors required to represent X. The individual elements of the decomposed tensor are expressed as

(2) $$x_{i,j,k}\approx \sum_{r=1}^{R}a_{ir}b_{jr}c_{kr},$$

where 1≤i≤I, 1≤j≤J, 1≤k≤K.

### 2.2. Proposed Approaches

The prediction of drug–gene–disease associations can be framed as a tensor completion problem involving the drug–gene–disease tensor denoted as $X\in R^{I\times J\times K}$ where *I*, *J*, and *K* indicate the number of drugs, genes, and diseases, respectively. When drug *i* is used to treat disease *k*, drug *i* binds to gene *j*, and disease *k* is caused by gene *j*, a triple link is considered to exist among drug *i*, gene *j*, and disease *k*, thereby setting the corresponding element $x_{ijk}$ of the tensor to 1. Initially, the remaining elements not associated with drug–gene–disease interactions are assigned a value of 0, and our goal was to estimate the scores for these other elements.

ID embedding vectors for drug *i*, gene *j*, and disease *k* are typically regarded as latent vectors for drugs, genes, and diseases, respectively [37]. Additional biological features pertaining to drugs, genes, and diseases can be provided as supplementary inputs. The drug–drug, gene–gene, and disease–disease similarities are utilized as extra information and normalized within the range of 0 to 1. Ultimately, the two sets of features, namely, the ID embedding and similarity vectors, are combined by concatenation as follows:

(3) $$dr_{i}=\left[\begin{array}{c} dr_{id_{i}}\\ dr_{sim_{i}} \end{array}\right],\;\;ge_{j}=\left[\begin{array}{c} ge_{id_{j}}\\ ge_{sim_{j}} \end{array}\right],\;\;di_{k}=\left[\begin{array}{c} di_{id_{k}}\\ di_{sim_{k}} \end{array}\right].$$

These combined vectors are taken as inputs of the fully connected neural network to convert the embeddings to a uniform size, $a_{i}\in R^{r}$, $b_{j}\in R^{r}$, and $c_{k}\in R^{r}$.

The proposed approach comprises two components: generalized tensor decomposition (GTD) and multi-layer perceptron (MLP). The GTD [36] extends CP decomposition to effectively capture the associations among drugs, genes, and diseases. Consequently, the embeddings obtained for drugs, genes, and diseases via the fully connected neural layers can be interpreted as latent vectors. The functions that map the drug–gene–disease associations can be defined as follows:

(4) $$\phi^{GTD}\left(a_{i},b_{j},c_{k}\right)=a_{i}⊙b_{j}⊙c_{k},$$

where ⊙ denotes the element-wise product of the vectors $a_{i}$, $b_{j}$, and $c_{k}$. These vectors are projected onto the output layer to create a tensor containing the estimated scores, as follows:

(5) $${\hat{X}}_{i,j,k}=\sigma \left(h^{T}\left(a_{i}⊙b_{j}⊙c_{k}\right)\right),$$

where σ is an activation function and *h* denotes a vector for edge weights in the output layer. This model was transformed into a CP decomposition by utilizing the identity function and uniform vector of 1 as σ and *h*, respectively. In other words, GTD represents an extension of CP decomposition to a nonlinear model, enabling it to effectively capture the complex structure of drug–gene–disease associations. In our approach, we employed the sigmoid function as σ to ensure that the output values lie within the range of 0 to 1, whereas *h* is learned from the data.

Although GTD effectively models drug–gene–disease associations, it does not fully capture the individual features of drugs, genes, and diseases, as latent vectors are not treated independently. To address this limitation, we incorporate an MLP model to independently learn drug, gene, and disease features. The multi-layer structure of the MLP is constructed as follows: initially, the latent vectors $a_{i}$, $b_{j}$, and $c_{k}$ corresponding to the drugs, genes, and diseases, respectively, are provided as inputs to the MLP.

(6) $$z_{dr}^{\left(0\right)}\;=\;\phi_{dr}^{\left(0\right)}\left(a_{i}\right)\;=\;a_{i},$$

(7) $$z_{ge}^{\left(0\right)}\;=\;\phi_{ge}^{\left(0\right)}\left(b_{j}\right)\;=\;b_{j},$$

(8) $$z_{di}^{\left(0\right)}\;=\;\phi_{di}^{\left(0\right)}\left(c_{k}\right)\;=\;c_{k}.$$

The operations performed in the first hidden layer are as follows: The outputs of the previous layers, $z_{dr}^{\left(0\right)}$, $z_{ge}^{\left(0\right)}$, and $z_{di}^{\left(0\right)}$, are considered as inputs multiplied by the weight matrices of the first layer, $W_{dr}^{\left(1\right)}$, $W_{ge}^{\left(1\right)}$, and $W_{di}^{\left(1\right)}$, respectively. In addition, the biases of the first layer, represented as $b_{dr}^{\left(1\right)}$, $b_{ge}^{\left(1\right)}$, and $b_{di}^{\left(1\right)}$, are added to activate this function.

(9) $$\phi_{dr}^{\left(1\right)}\left(z_{dr}^{\left(0\right)}\right)\;=\;\sigma_{dr}^{\left(1\right)}\left({W_{dr}^{\left(1\right)}}^{T}z_{dr}^{\left(0\right)}+\;b_{dr}^{\left(1\right)}\right),$$

(10) $$\phi_{ge}^{\left(1\right)}\left(z_{ge}^{\left(0\right)}\right)\;=\;\sigma_{ge}^{\left(1\right)}\left({W_{ge}^{\left(1\right)}}^{T}z_{ge}^{\left(0\right)}+\;b_{ge}^{\left(1\right)}\right),$$

(11) $$\phi_{di}^{\left(1\right)}\left(z_{di}^{\left(0\right)}\right)\;=\;\sigma_{di}^{\left(1\right)}\left({W_{di}^{\left(1\right)}}^{T}z_{di}^{\left(0\right)}+\;b_{di}^{\left(1\right)}\right).$$

These operations are iteratively performed until reaching the *L*-th layer.

(12) $$\phi_{dr}^{\left(L\right)}\left(z_{dr}^{\left(L-1\right)}\right)\;=\;\sigma_{dr}^{\left(L\right)}\left({W_{dr}^{\left(L\right)}}^{T}z_{dr}^{\left(L-1\right)}+\;b_{dr}^{\left(L\right)}\right),$$

(13) $$\phi_{ge}^{\left(L\right)}\left(z_{ge}^{\left(L-1\right)}\right)\;=\;\sigma_{ge}^{\left(L\right)}\left({W_{ge}^{\left(L\right)}}^{T}z_{ge}^{\left(L-1\right)}+\;b_{ge}^{\left(L\right)}\right),$$

(14) $$\phi_{di}^{\left(L\right)}\left(z_{di}^{\left(L-1\right)}\right)\;=\;\sigma_{di}^{\left(L\right)}\left({W_{di}^{\left(L\right)}}^{T}z_{di}^{\left(L-1\right)}+\;b_{di}^{\left(L\right)}\right).$$

Finally, the tensor containing the scores for drug–gene–disease associations can be derived by taking the element-wise product of the three components.

(15) $${\hat{X}}_{ijk}\;=\;\sigma \left(h^{T}\left(\phi_{dr}^{\left(L\right)}\left(z_{dr}^{\left(L-1\right)}\right)\;*\;\phi_{ge}^{\left(L\right)}\left(z_{ge}^{\left(L-1\right)}\right)\;\;*\;\phi_{di}^{\left(L\right)}\left(z_{di}^{\left(L-1\right)}\right)\;\right)\right).$$

The MLP structure follows a tower pattern characterized by multiple layers, where the bottom layer has the greatest number of neurons and the subsequent layers have gradually decreasing numbers of neurons. Drug, gene, and disease features learned independently through the MLP are regarded as latent vectors representing drugs, genes, and diseases, respectively.

As mentioned earlier, GTD extends CP decomposition to model drug–gene–disease associations, whereas MLP identifies complex features of drugs, genes, and diseases. In this study, we propose a novel approach that combines the elements of the GTD and MLP. This model integrates an input consisting of ID embedding vectors and similarities and applies both GTD and MLP. The resulting drug, gene, and disease latent vectors from the GTD and MLP are merged to generate the final latent vectors and predict the drug–gene–disease associations. By leveraging GTD for modeling associations and MLP for learning features, this approach offers greater flexibility and non-linearity than traditional tensor decomposition methods, allowing it to capture complex drug–gene–disease associations.

## 3. Data

For our experiments, we created drug–gene–disease tensors based on drug–disease, drug–gene, and disease–gene associations. We also constructed similarity networks using biological features associated with drugs, genes, and diseases. The tensors and similarity networks were acquired as described below.

### 3.1. Pairwise Associations

First, we used drug–disease associations from the Cdataset [24], a widely recognized benchmark dataset for validating computational drug repositioning methods. This dataset combines data from the Fdataset [38] and the DNdataset [39], containing 663 distinct drugs from DrugBank [40], 409 diseases from Online Mendelian Inheritance in Man (OMIM) [41] and 2352 known drug–disease associations. Next, we extracted drug–gene associations from the Comparative Toxicogenomic Database (CTD) [42], which provides not only interactions between chemicals and genes, specifically DTIs, but also offers insights into their associations with diseases. The CAS numbers of the drugs were mapped to gene symbols. Finally, we obtained disease–gene associations from OMIM, a comprehensive resource offering extensive information on human genes and phenotypes, including various disorders.

### 3.2. Drug Similarity Networks

To construct drug similarity networks as supplementary information, we used DrugBank 5.0 [40], which provides a wide range of drug features, including chemical structures, anatomical therapeutic chemical classification system (ATC) codes [43], targets, and side effects. This comprehensive database encompasses both drugs approved by Food and Drug Administration (FDA) and experimental drugs undergoing approval procedures. We considered both approved and experimental drugs for drug similarity networks, as drug repositioning aims to identify new therapeutic indications for not only already commercialized drugs, but also those that were not brought to the market due to insufficient clinical efficacy. In our experiment, we used two distinct drug similarity networks based on chemical structures and ATC codes.

Drugs with similar chemical structures tend to exhibit similar features. The chemical structures of the drugs are stored in DrugBank using the simplified molecular input line entry system (SMILES) notation [44], which represents structures as strings. We used this information to calculate drug–drug similarities by employing a chemistry development kit (CDK) [45], an open-source library specializing in structural chemistry and bioinformatics. From the drugs included in the Cdataset, a drug similarity network was established based on the chemical structures of 659 drugs with available SMILES information from DrugBank.

The ATC code, a drug classification system devised by the World Health Organization (WHO), classifies drugs into the categories from five levels. We considered two drugs to be similar if they shared the same code at any classification level. The similarity between drugs $d_{i}$ and $d_{j}$ based on the *k*-th level of the ATC codes was defined as follows [46]:

(16) $$S_{k}\left(d_{i},\;d_{j}\right)\;=\;\frac{{ATC}_{k}\left(d_{i}\right)\;\cap \;{ATC}_{k}\left(d_{j}\right)}{{ATC}_{k}\left(d_{i}\right)\;\cup \;{ATC}_{k}\left(d_{j}\right)}.$$

${ATC}_{k}$ represents a set of codes corresponding to the *k*-th level of all ATC codes assigned to a particular drug. It is important to note that a single drug can be associated with multiple ATC codes. Finally, the similarity between drugs was calculated by averaging their similarities across all levels.

(17) $${sim}_{atc}\left(d_{i},\;d_{j}\right)\;=\;\frac{\sum_{k=1}^{n}S_{k}\left(d_{i},\;d_{j}\right)}{n},$$

where n=5 because the ATC codes are composed of five levels. From the drugs present in the Cdataset, a drug similarity network was constructed based on ATC codes, involving 636 drugs for which ATC code information was available in DrugBank.

### 3.3. Gene Similarity Networks

An ontology is a hierarchical arrangement of terms, in which those with more specific meanings are assigned to subsets. Typically, this organization takes the form of a directed acyclic graph, with edges representing parent–child relationships between the terms. Gene Ontology (GO) [47] is the most widely referenced ontology in this field. It comprises terms that serve as biological descriptions with genes annotated to their corresponding terms. By leveraging the GO structure and annotations, we can classify the biological roles of these genes.

Assuming that similar genes perform similar functions, we can measure the similarity between genes by assessing the semantic similarity between the GO terms to which they are annotated. We employed a semantic similarity metric [48] to calculate the ratio of information content for common GO terms as follows:

(18) $${sim}_{T}\left(C_{1},\;C_{2}\right)=\;\frac{\sum_{C_{i}\in A_{t}\left(C_{1}\right)\cap A_{t}\left(C_{2}\right)}log\;P(C_{i})}{\sum_{C_{j}\in A_{t}\left(C_{1}\right)\cup A_{t}\left(C_{2}\right)}log\;P(C_{j})},$$

where $C_{1}$ and $C_{2}$ are GO terms, $A_{t}\left(C_{i}\right)$ denotes the set of ancestral terms of $C_{i}$, $P(C_{i})$ signifies the proportion of genes annotated to $C_{i}$, and $-log\;P(C_{i})$ indicates the information content of $C_{i}$. Given that genes can be annotated to multiple terms, we computed the similarity between two genes using the best matching average of all the term pairs in which the genes were annotated.

(19) $$sim\left(g_{1},\;g_{2}\right)\;=\;\frac{\sum_{C_{i}\in T\left(g_{1}\right)}{max}_{C_{j}\in T\left(g_{2}\right)}{sim}_{T}\left(C_{i},C_{j}\right)\;+\;\sum_{C_{j}\in T\left(g_{2}\right)}{max}_{C_{i}\in T\left(g_{1}\right)}{sim}_{T}\left(C_{i},C_{j}\right)}{|T\left(g_{1}\right)|\;+\;|T\left(g_{2}\right)|\;},$$

where T(g) represents the set of GO terms to which the gene *g* is annotated. This formula was applied to the sub-ontologies of biological processes and molecular functions in GO. To enhance the data reliability, annotations with the evidence code of IEA or qualifiers indicating “not” were excluded from this experiment.

A gene similarity network was generated by retrieving protein–protein interactions (PPIs) from BioGRID [49]. Subsequently, the similarities of interacting pairs in the refined set of PPIs were computed after deleting redundant links and self-loops. To further address network sparsity, genes with fewer than 10 drug–gene or disease–gene associations were excluded. Finally, a similarity network consisting of 7824 genes was constructed.

### 3.4. Disease Similarity Networks

Similar to the gene similarity network, we created a disease similarity network by calculating semantic similarities between diseases using the Human Phenotype Ontology (HPO) [50] and its annotations. The HPO is an ontology that classifies phenotypes associated with human diseases and mutations. Assuming that similar diseases exhibit shared symptoms, the similarity between diseases was measured by the semantic similarity between the HPO terms to which the diseases were annotated. Formula (18) was employed to calculate semantic similarity and Formula (19) was used to determine the similarity between the diseases. For semantic similarity, we focused on sub-terms of “Phenotypic Abnormality” in HPO and excluded diseases annotated with the evidence code of IEA. The HPO includes annotations of more than 50,000 diseases from OMIM [41], OrphaNet [51], and DECIPHER [52]. For this experiment, we selected diseases from OMIM. The resulting disease similarity network consisted of 285 diseases from the Cdataset. Table 1 provides an overview of the number of nodes and edges in the drug, gene, and disease similarity networks.

### 3.5. Drug–Gene–Disease Tensors

Based on the drug features of chemical structures and ATC codes, we generated two distinct drug–gene–disease tensors. The tensor using the chemical structures of drugs contained 252 drug–gene–disease triple associations, including 659 drugs, 7824 genes, and 285 diseases. In contrast, the tensor based on the ATC codes of drugs contained 251 drug–gene–disease triple associations, involving 636 drugs, 7824 genes, and 285 diseases. Consequently, both the tensors exhibited similar levels of sparsity. Please refer to Table 2 for the drug–disease, drug–gene, and disease–gene pairwise associations employed in creating drug–gene–disease tensors.

## 4. Results

Experiments for drug–gene–disease triple-association prediction were conducted separately on the drug and disease sides. On the drug side, we examined the prediction of triple associations for a novel drug without any known drug–disease or drug–gene associations. This experiment involved the prediction of genes interacting with the new drug and diseases resulting from mutations in these genes. Conversely, on the disease side, we tested the prediction of triple associations for a new disease without any known drug–disease or disease–gene associations. The objective was to predict the genes responsible for the new disease and drugs targeting these genes. To evaluate the performance of the drug–gene–disease association prediction, we conducted a 10-fold cross-validation for each side. The folds were partitioned to ensure an even distribution of known triple associations.

The drug–gene–disease tensor X is typically very sparse owing to the limited number of drug–disease, drug–gene, and disease–gene associations. This sparsity causes a severe imbalance between positive and negative samples, which can hinder model learning. To address this issue, we introduced negative samples by randomly selecting 10 unlinked elements between the drugs, genes, and diseases for each positive sample. This strategy resulted in a positive-to-negative sample ratio of 1 to 10.

The performance of the predictive model was assessed using two evaluation metrics: the area under the ROC curve (AUC) and normalized discrete cumulative gain (NDCG). The ROC curve was plotted by tracking the true-positive rate as the false-positive rate increases, and the area below the ROC curve is an indicator of the predictive accuracy of the binary classification models. The true-positive rate represents the proportion of samples correctly predicted to be positive among all positive samples, whereas the false-positive rate represents the proportion of samples incorrectly predicted to be positive among all negative samples.

The NDCG is an evaluation metric used in ranking-based recommendation systems. It assesses the quality of the top n predicted drug–gene–disease associations by assigning higher weights to those that are ranked more prominently. NDCG was calculated by normalizing the discounted cumulative gain (DCG) to ideal DCG (IDCG). The DCG was determined based on the relevance scores and rankings of the recommendation results.

(20) $$DCG=\;\sum_{i=1}^{n}\frac{{rel}_{i}}{{log}_{2}\left(i+1\right)},$$

where rel represents the relevance score assigned to the recommended results and *n* denotes the total number of ranked results used for evaluation. IDCG represents the DCG value when all recommended results are perfectly ordered. Both the AUC and NDCG values range between 0 and 1. Values closer to 1 indicate better predictive accuracy of the models.

### 4.1. Predicting Drug–Gene–Disease Triple Associations

First, we evaluated the performance of predicting drug–gene–disease triple associations on the drug side. This involved predicting associations for each drug without any prior knowledge of drug–disease or drug–gene associations. For the experimental dataset, we created a balanced set by randomly sampling 10 negative samples for each positive sample. To facilitate learning, positive and negative association pairs were divided into 10 folds based on the drugs. One fold was used for validation to terminate early if the performance did not improve, preventing the model from overfitting. Based on the prediction scores of the selected drug–disease pairs, we assessed the performance of the GTD, MLP, and their ensemble models using AUC and NDCG@n. The NDCG@n metric evaluates the accuracy of the top n associations based on their prediction scores. The results of the proposed approaches were compared with those of NTD-DR [34], which is a conventional tensor decomposition method for drug repositioning.

The predictive accuracies on the drug side with respect to the AUC and NDCG@n scores with n values of 1, 3, 5, and 10 are presented in Table 3. The proposed ensemble model consistently exhibited the highest predictive accuracy across both drug features as additional information. NTD-DR showed a lower overall accuracy than the proposed approach. When comparing the GTD and MLP, MLP performed better than GTD in terms of AUC. However, in terms of NDCG, the performance varied depending on the drug features. Specifically, when drug–drug similarity was measured using chemical structures, GTD achieved higher NDCG values than MLP. In contrast, the MLP, which learns drug, gene, and disease features individually, performed better than the GTD when similarity based on ATC codes was used.

**Figure 1:**
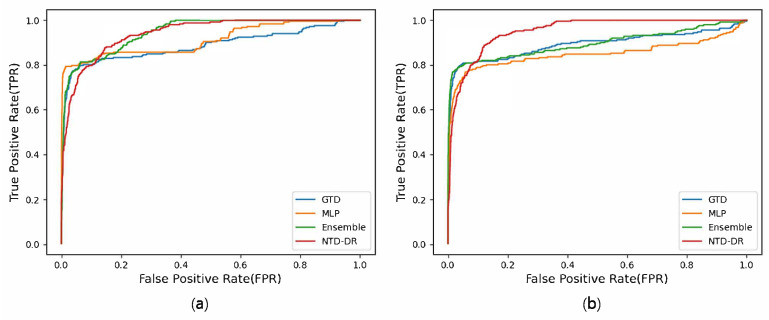
ROC curves of drug–gene–disease triple association prediction experiments on the drug side using the networks based on (**a**) chemical structures and (**b**) ATC codes of drugs.

Figure 1 shows the ROC curves of the drug–gene–disease association prediction experiments for a novel drug. Figure 1a shows the results from the drug similarity networks using chemical structures, while Figure 1b shows the results from the ATC code-based drug similarity networks. In both graphs, the proposed ensemble model and MLP exhibit a steep increase in the true-positive rate and are positioned in the upper-left corner. In contrast, NTD-DR and GTD show gradual increases in the true-positive rate as the false-positive rate increases.

Next, we evaluated the predictive performance of the drug–gene–disease triple associations on the disease side. This involved predicting associations for each disease without any prior knowledge of drug–disease or disease–gene associations. Similar to the drug side prediction, we divided the positive and negative association pairs into 10 folds based on diseases for the learning process. The AUC and NDCG results for the predictive outcomes on the disease side are presented in Table 4. Consistent with the findings in Table 3, the proposed ensemble model demonstrated a superior performance except for the AUC results from the ATC code-based drug similarity network. Although NTD-DR showed the highest AUC value, its NDCG results decreased. In terms of the NDCG index, the MLP ranked second after the ensemble method. These results can be attributed to the fact that AUC reflects classification performance, whereas NDCG considers the ranking of the predicted outcomes. In other words, NTD-DR performed well in determining the presence or absence of drug–gene–disease associations in terms of classification, whereas the MLP excelled in determining the ranking of these associations.

**Figure 2:**
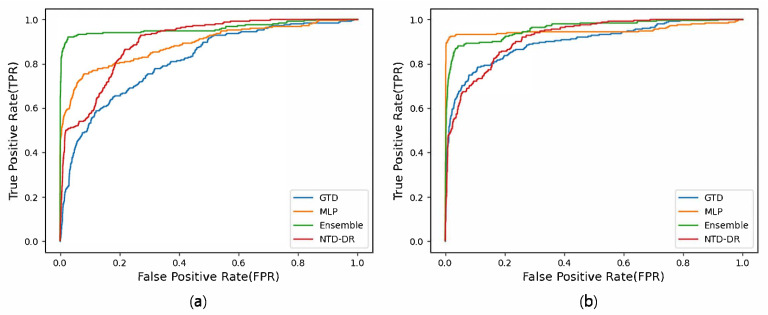
ROC curves of drug–gene–disease triple association prediction experiments on the disease side using the networks based on (**a**) chemical structures and (**b**) ATC codes of drugs.

Figure 2 illustrates the ROC curves of the drug–gene–disease association prediction experiments for novel diseases, with (a) representing the results using drug similarity networks based on chemical structures and (b) representing the results using ATC code-based drug similarity networks. Similar to the findings in Figure 1, NTD-DR shows a gradual increase in the true-positive rate as the false-positive rate increases. In contrast, the proposed ensemble model demonstrated a rapid increase in the true-positive rate. These results provide evidence that the ensemble model attained the highest NDCG values, as listed in Table 4.

When comparing the prediction performance between the drug and disease sides, we observed that triple associations were generally better predicted for new drugs than for new diseases. This is primarily due to the larger number of drugs with available drug–gene–disease triple associations compared to the number of diseases. Moreover, when comparing the prediction performance between the two drug features used, the drug similarity network based on ATC codes showed a higher predictive accuracy on both the drug and disease sides than the network based on chemical structures. Finally, in all cases, the performance of the ensemble model was significantly improved over GTD. As the most significant result of this study, the accuracy of drug–gene–disease triple association prediction was enhanced by employing MLP to reflect and learn the distinct features of drugs, genes, and diseases.

### 4.2. Predicting Drug–Disease Pairwise Associations

The decomposition of drug–disease, drug–gene, and disease–gene matrices yields latent vectors for drugs, genes, and diseases, which can be used to identify pairwise associations between them. The latent vectors obtained through the prediction of drug–gene–disease triple associations, as conducted in previous experiments, also provide evidence of the associations between drugs, genes, and diseases. Consequently, pairwise associations among drugs, genes, and diseases can be predicted by taking the inner product of their corresponding latent vectors obtained from the tensor decomposition of drug–gene–disease associations.

First, drug–disease association prediction was performed using drug and disease latent vectors obtained through the prediction of drug–gene–disease triple associations. In terms of predictive accuracy, this approach was compared to several existing computational drug repositioning methods, namely MBiRW [24], MSBMF [28], OMC [31], and deepDR [26]. Table 5 presents the AUC and NDCG results of the drug–disease association prediction for new drugs without any prior knowledge of drug–disease or drug–gene associations. Overall, OMC, a previous matrix factorization method, and the proposed ensemble model demonstrated the best performance in this evaluation. Unlike other drug repositioning methods, OMC incorporates not only drug–disease associations but also drug–gene and disease–gene associations to capture the features of genes that are targeted by the drug and cause the disease. This result suggests that accurate drug repositioning relies not only on drug features but also on gene and disease features. The deep learning algorithm, deepDR, exhibited relatively poor performance as it did not utilize a disease similarity network, resulting in insufficient incorporation of disease features.

### 4.3. Predicting Drug–Gene Pairwise Associations

Subsequently, in a manner similar to drug–disease association prediction, drug–gene associations were predicted using latent vectors for drugs and genes obtained through drug–gene–disease triple association prediction. The predictive accuracy of the proposed approach was compared with that of previous DTI prediction methods, specifically NetLapRLS [11], BLM-NII [9], and MSCMF [12]. Table 6 presents the AUC and NDCG results of the drug–gene association prediction for new drugs without any prior knowledge of drug–disease or drug–gene associations. Overall, the proposed ensemble model outperformed the other methods in terms of predictive accuracy, regardless of the drug features employed. GTD also demonstrated a strong performance, particularly in NDCG. GTD and MLP generally showed similar trends to the ensemble model, suggesting that incorporating drug–disease and disease–gene associations, along with disease features, contributes to improved accuracy in predicting drug–gene associations. Among the previous methods, NetLapRLS achieved relatively favorable results in terms of both the AUC and NDCG, particularly when larger values of *n* were considered.

### 4.4. Predicting Disease–Gene Pairwise Associations

By prioritizing genes associated with a specific disease based on the prediction scores of disease–gene associations, candidate genes responsible for the disease can be identified. The prediction of disease–gene associations is accomplished using latent vectors representing diseases and genes obtained through drug–gene–disease triple association prediction. Table 7 presents the AUC and NDCG results of the disease–gene association prediction for GTD, MLP, the proposed ensemble model, and existing disease–gene prioritization methods, namely PRINCE [20] and HerGePred [21]. The results demonstrated that the ensemble model, which leverages drug–gene and drug–disease associations, outperformed the other methods in terms of predictive accuracy.

### 4.5. Hyperparameter Tuning

The rank in the tensor decomposition corresponds to the number of latent factors required to represent the tensor and determines the size of the latent matrix. Generally, a lower rank yields a simpler decomposition result but with reduced accuracy. Conversely, a higher rank produces a more accurate decomposition result but increases the computational complexity and risk of overfitting. Because the optimal rank can vary depending on the size of the data and quality of the features, it is considered a hyperparameter to be chosen.

**Figure 3:**
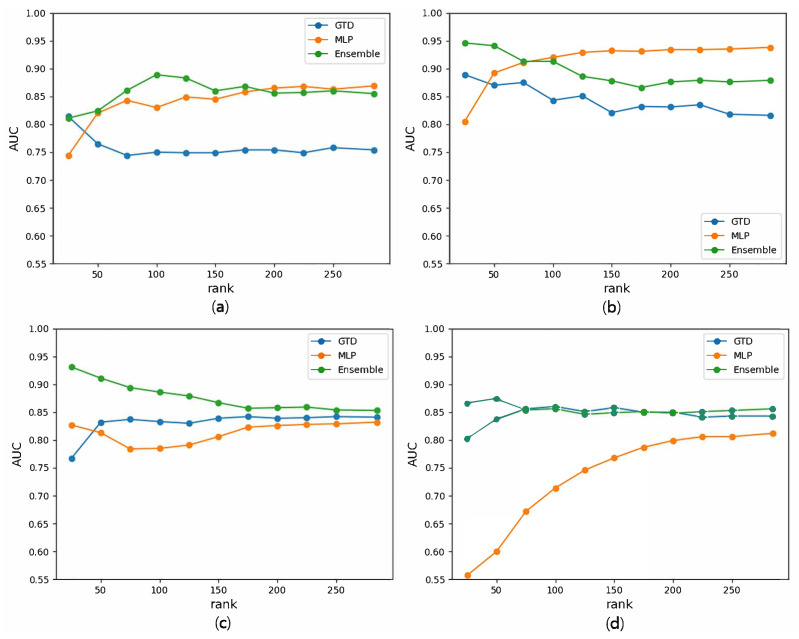
AUC changes with respect to the rank for the prediction of drug–gene–disease triple associations using the networks based on chemical structures in (**a**,**b**), using the networks based on ATC codes in (**c**,**d**), on the drug side in (**a**,**c**), and on the disease side in (**b**,**d**).

Figure 3 illustrates the AUC changes according to the rank for the prediction of drug–gene–disease triple associations. Figure 3a,b present the results when utilizing chemical structures as a drug feature, whereas Figure 3c,d display the results using the ATC code-based drug similarity network. Also, Figure 3a,c show the prediction results for new drugs, whereas Figure 3b,d show the prediction results for new diseases. It can be observed that GTD and the ensemble model generally tend to overfit as the rank increases, resulting in poor performance. However, MLP improves the performance as the rank increases, although the rate of improvement gradually diminishes. The rank employed in the previous experiment was determined to be the highest performing rank because no method for identifying the optimal rank in tensor decomposition was available.

### 4.6. Balancing Training Datasets

The drug–gene–disease tensor used in this experiment exhibited a high level of sparsity, resulting in an imbalance between the positive and negative samples. To address this issue, we randomly selected 10α negative samples per positive sample, where α represents the proportion of negative to positive samples. Increasing the value of α further exacerbated this imbalance. It is important to note that achieving a balance in the training dataset between positive and negative samples can impact the predictive accuracy of the model, thus necessitating the determination of an appropriate α.

**Figure 4:**
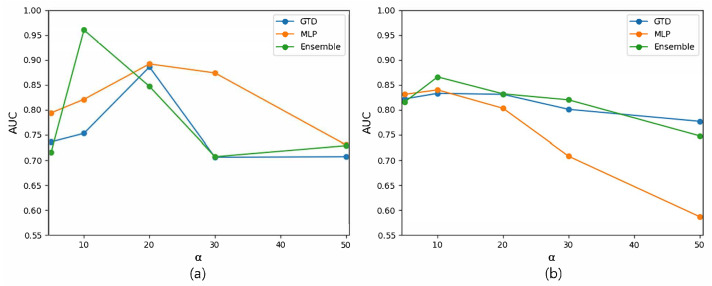
AUC changes with respect to the proportion of negative samples to a positive for the prediction of drug–gene–disease triple associations using the networks based on (**a**) chemical structures and (**b**) ATC codes of drugs.

Figure 4 presents the experimental results obtained while exploring the drug–gene–disease triple association prediction to identify the optimal value of α using the drug similarity network of chemical structures (Figure 4a) and the network based on the ATC code (Figure 4b). When α was less than 10, GTD exhibited inaccurate predictions because it did not adequately consider the negative samples in the tensor. When α was set to 10, GTD, MLP, and the ensemble model demonstrated the best predictive performance. However, as α exceeded 10, the accuracy of MLP significantly decreased. This indicates that the model became overfitted to the negative samples as α increased. Consequently, the optimal α for drug–gene–disease triple association prediction was determined to be 10.

## 5. Discussion and Conclusions

Drug repositioning provides considerable benefits in terms of safety, time, and cost, by identifying new therapeutic indications for existing drugs. Computational approaches are highly anticipated because of their effectiveness in inferring potential associations between drugs and diseases. Over the past few years, numerous network-based drug repositioning methods have been proposed. Moreover, since drugs achieve their therapeutic effects by targeting specific genes, it is crucial to identify the proteins targeted by drugs and the genes implicated in diseases to facilitate successful drug repositioning. Consequently, simultaneous research has been conducted on drug–target interaction prediction and disease–gene prioritization methods to complement drug repositioning efforts.

In this study, we introduced a novel model that integrates GTD and MLP to identify triple associations among drugs, genes, and diseases. Our approach combines ID embedding vectors for drugs, genes, and diseases with biological similarities to facilitate learning. Through extensive experiments, we demonstrated that our proposed ensemble model surpassed the traditional tensor decomposition method in accurately predicting triple associations. This improvement can be attributed to the combination of GTD, which models drug–gene–disease associations, and MLP, which learns the individual features of drugs, genes, and diseases. As a result, our model captured complex associations among drugs, genes, and diseases more effectively than conventional element-wise multiplication in tensor decomposition.

Moreover, we leveraged the latent vectors for drugs, genes, and diseases obtained from the drug–gene–disease association prediction experiments to extend our approach for predicting drug–disease, drug–gene, and disease–gene associations. The proposed ensemble model consistently demonstrated superior performance in pairwise association predictions. Notably, previous methods that incorporated features from all three components, drugs, genes, and diseases, also yielded accurate results. These findings highlight the interactions among drugs, genes, and diseases, emphasizing the importance of considering genes in drug repositioning, diseases in DTI prediction, and drugs in disease-causing gene prioritization. Additionally, our proposed model enables the simultaneous prediction of drug–disease, drug–gene, and disease–gene associations through drug–gene–disease association prediction. Consequently, our ensemble model achieved fast performance in these tasks compared with existing methods that predict each type of pairwise association separately or the conventional tensor decomposition approach, which requires learning from large tensors.

This study has significant implications for the field of medical science. Possible implications include not only expanding the range of treatment for diseases but also identifying novel drug targets and disease mechanisms. Genetic information regarding therapeutic targets for drug repositioning can greatly contribute to personalized medicine by tailoring treatments based on an individual’s genetic makeup, thus minimizing adverse drug reactions and maximizing therapeutic efficacy. Moreover, leveraging genetic information allows for the assessment of disease risks and the implementation of preventive measures for specific conditions. Ultimately, these advancements will enable the development of more targeted and effective healthcare approaches.

## Figures and Tables

**Table 1:** Number of nodes and edges in the drug, gene, and disease similarity networks.

		Number of Nodes	Number of Edges
Drug network	chemical structures	659	216,308
	ATC codes	636	161,332
Disease network		7824	80,849
Gene network		285	18,392

**Table 2:** Number of nodes and edges of drug–disease, drug–gene, and disease–gene pairwise associations to create drug–gene–disease tensors based on two distinct drug features, chemical structures, and ATC codes.

		Number of	Number of	Number of	Number of
		Drugs	Genes	Diseases	Associations
Chemical structures	drug–disease	615	-	285	1728
	drug–gene	452	7824	-	137,054
	disease–gene	-	168	290	361
ATC codes	drug–disease	593	-	282	1681
	drug–gene	438	7824	-	134,481
	disease–gene	-	168	290	361

**Table 3:** Accuracy of predicting drug–gene–disease triple associations on the drug side.

Method	Networks Constructed by Chemical Structures					Networks Constructed by ATC Codes				
	AUC	NDCG@1	NDCG@3	NDCG@5	NDCG@10	AUC	NDCG@1	NDCG@3	NDCG@5	NDCG@10
GTD	0.814	0.737	0.801	0.828	0.857	0.899	0.798	0.841	0.857	0.880
MLP	0.888	0.632	0.689	0.723	0.779	**0.950**	0.872	0.905	0.910	0.920
Ensemble	**0.960**	**0.905**	**0.921**	**0.940**	**0.945**	**0.959**	**0.936**	**0.946**	**0.951**	**0.960**
NTD-DR	0.887	0.853	**0.914**	0.928	0.930	0.918	0.894	0.918	**0.941**	0.943

**Table 4:** Accuracy of predicting drug–gene–disease triple associations on the disease side.

Method	Networks Constructed by Chemical Structures					Networks Constructed by ATC Codes				
	AUC	NDCG@1	NDCG@3	NDCG@5	NDCG@10	AUC	NDCG@1	NDCG@3	NDCG@5	NDCG@10
GTD	0.886	0.656	0.753	0.768	0.805	0.891	0.787	0.842	0.862	0.882
MLP	0.912	**0.869**	0.870	0.869	0.883	0.871	0.902	0.904	0.912	0.918
Ensemble	**0.947**	**0.869**	**0.886**	**0.911**	**0.922**	0.895	**0.918**	**0.921**	**0.933**	**0.947**
NTD-DR	**0.941**	0.705	0.800	0.817	0.825	**0.934**	0.770	0.849	0.842	0.857

**Table 5:** Accuracy of predicting drug–disease pairwise associations on the drug side.

Method	Networks Constructed by Chemical Structures					Networks Constructed by ATC Codes				
	AUC	NDCG@1	NDCG@3	NDCG@5	NDCG@10	AUC	NDCG@1	NDCG@3	NDCG@5	NDCG@10
GTD	0.766	0.305	0.424	0.517	0.606	0.854	0.415	0.538	0.608	0.663
MLP	0.812	0.316	0.438	0.491	0.598	0.848	0.426	0.586	0.643	0.710
Ensemble	**0.835**	**0.379**	**0.546**	**0.603**	**0.669**	0.874	0.500	0.643	0.702	0.756
MBiRW	0.707	0.238	0.400	0.478	0.539	0.867	0.568	0.699	0.740	0.788
MSBMF	0.715	0.290	0.460	0.521	0.573	0.823	0.530	0.685	0.703	0.756
OMC	0.806	**0.384**	**0.564**	**0.609**	**0.665**	**0.902**	**0.592**	**0.734**	**0.769**	**0.806**
deepDR	0.662	0.038	0.039	0.098	0.195	0.565	0.171	0.267	0.310	0.415

**Table 6:** Accuracy of predicting drug–gene pairwise associations on the drug side.

Method	Networks Constructed by Chemical Structures					Networks Constructed by ATC Codes				
	AUC	NDCG@1	NDCG@3	NDCG@5	NDCG@10	AUC	NDCG@1	NDCG@3	NDCG@5	NDCG@10
GTD	0.595	0.747	**0.792**	**0.803**	0.818	0.652	0.904	**0.902**	**0.883**	**0.896**
MLP	0.580	**0.768**	**0.781**	0.786	0.816	0.653	0.894	0.881	0.873	0.880
Ensemble	**0.619**	**0.779**	**0.796**	**0.809**	0.828	0.681	**0.926**	**0.918**	**0.901**	**0.904**
NetLapRLS	**0.610**	0.491	0.721	**0.796**	**0.878**	**0.709**	0.501	0.730	0.810	**0.889**
BLM-NII	0.567	0.320	0.498	0.578	0.735	0.514	0.345	0.518	0.618	0.760
MSCMF	**0.613**	0.304	0.422	0.485	0.690	0.517	0.283	0.398	0.502	0.688

**Table 7:** Accuracy of predicting disease–gene pairwise associations on the disease side.

Method	Networks Constructed by Chemical Structures					Networks Constructed by ATC Codes				
	AUC	NDCG@1	NDCG@3	NDCG@5	NDCG@10	AUC	NDCG@1	NDCG@3	NDCG@5	NDCG@10
GTD	0.884	0.656	0.776	0.807	0.844	0.879	0.557	0.708	0.748	0.778
MLP	0.894	**0.869**	**0.882**	**0.893**	**0.908**	0.793	**0.852**	0.871	0.886	0.894
Ensemble	**0.926**	**0.852**	**0.884**	**0.905**	**0.919**	**0.924**	**0.869**	**0.912**	**0.917**	**0.924**
PRINCE	0.633	0.363	0.497	0.581	0.704	0.637	0.371	0.493	0.567	0.703
HerGePred	0.681	0.330	0.561	0.631	0.718	0.677	0.393	0.567	0.642	0.741

## Data Availability

https://ads.yonsei.ac.kr/Ensemble/ (accessed on 15 June 2023).
